# Supplementary material for: EnzML: multi-label prediction of enzyme classes using InterPro signatures
Source: BMC Bioinformatics. 2012 Apr 25;13:61. doi: 10.1186/1471-2105-13-61 (PMC3483700; doi:10.1186/1471-2105-13-61)
Supplement: Addtional file 5 — The Java code to format the data files, evaluate and predict. The file enzml_java_code.tar.gz contains the Java code used to format database data to ARFF and XML formats, to execute cross and train-test (jackknife) evaluations and to record evaluation results to database. More information is included in the readme.txt file and the Javadoc files. The code can be used with a MySQL database. To use a different database software, other JDBC drivers might be required. [file 1471-2105-13-61-S5.gz › java_code/enzml2011/doc/uk/ac/ed/inf/enzml/mulan/attributesfilter/ClassEnrichedArff_old.html]

ClassEnrichedArff\_old


---


|  |  |  |
| --- | --- | --- |
| **Overview** \| **Package** \| **Class** \| **Use** \| **Tree** \| **Deprecated** \| **Index** \| **Help** | |  |
| **PREV CLASS**   NEXT CLASS | **FRAMES**    **NO FRAMES**     **All Classes** |  |
| SUMMARY: NESTED \| FIELD \| CONSTR \| METHOD | DETAIL: FIELD \| CONSTR \| METHOD |  |


---


## uk.ac.ed.inf.enzml.mulan.attributesfilter Class ClassEnrichedArff\_old

```
java.lang.Object
  uk.ac.ed.inf.utils.Initialised
      uk.ac.ed.inf.enzml.weka.Arff
          uk.ac.ed.inf.enzml.mulan.MulanArff
              uk.ac.ed.inf.enzml.mulan.attributesfilter.ClassEnrichedArff_old
```

**All Implemented Interfaces:**: uk.ac.ed.inf.utils.Initialisable

---

``` public class ClassEnrichedArff_old extends MulanArff ```

An ARFF taking its classes from an external query. In practice, this is used
to generate a training arff that includes also the test set class labels.
This allows the new test set to keep its full set of classes and still be
compatible with this new train set for a train/test prediction. It also
ensures the new test set can keep all its original classes in order to be
correctly evaluated in a train/test evaluation.

**Version:**
:   19 May 2011

**Author:**
:   Luna De Ferrari luna.deferrari-at-ed.ac.uk

---

**Field Summary**

**Fields inherited from class uk.ac.ed.inf.utils.Initialised**

`m_initialisationIsCorrect`


**Constructor Summary**

`ClassEnrichedArff_old(java.lang.String dataPropsFileName,
java.lang.String resultsPropsFileName,
AttributesFilter filterManager)`


| **Method Summary** | |
| --- | --- |
| `AttributesFilter` | `getFilterManager()` |

**Methods inherited from class uk.ac.ed.inf.enzml.mulan.MulanArff**

`getDataSetManager, getXmlPath, getXMLpathFromArffPath, main, run, run, setDataSetManager`

**Methods inherited from class uk.ac.ed.inf.enzml.weka.Arff**

`generateAndWriteArffFile, generateArff, getArffPath, getArffRecordId, getDataArffProperties, getDataDbManager, getDataPropsFilePath, getDbConn, getDbReader, getResultsDbManager, loadAndGenerateArff, setArffRecordId`

**Methods inherited from class uk.ac.ed.inf.utils.Initialised**

`initialisationIsCorrect, setInitialisationIsCorrect`

**Methods inherited from class java.lang.Object**

`equals, getClass, hashCode, notify, notifyAll, toString, wait, wait, wait`

**Constructor Detail**

### ClassEnrichedArff\_old

```
public ClassEnrichedArff_old(java.lang.String dataPropsFileName,
                             java.lang.String resultsPropsFileName,
                             AttributesFilter filterManager)
```


**Method Detail**

### getFilterManager

```
public AttributesFilter getFilterManager()
```


---


|  |  |  |
| --- | --- | --- |
| **Overview** \| **Package** \| **Class** \| **Use** \| **Tree** \| **Deprecated** \| **Index** \| **Help** | |  |
| **PREV CLASS**   NEXT CLASS | **FRAMES**    **NO FRAMES**     **All Classes** |  |
| SUMMARY: NESTED \| FIELD \| CONSTR \| METHOD | DETAIL: FIELD \| CONSTR \| METHOD |  |


---
